# Circulating Plasma MicroRNA-208a as Potential Biomarker of Chronic Indeterminate Phase of Chagas Disease

**DOI:** 10.3389/fmicb.2018.00269

**Published:** 2018-03-06

**Authors:** Leandra Linhares-Lacerda, Alessandra Granato, João Francisco Gomes-Neto, Luciana Conde, Leonardo Freire-de-Lima, Elisangela O. de Freitas, Celio G. Freire-de-Lima, Shana P. Coutinho Barroso, Rodrigo Jorge de Alcântara Guerra, Roberto C. Pedrosa, Wilson Savino, Alexandre Morrot

**Affiliations:** ^1^Departamento de Imunologia, Instituto de Microbiologia, Universidade Federal do Rio de Janeiro, Rio de Janeiro, Brazil; ^2^Instituto Oswaldo Cruz, Fundação Oswaldo Cruz (FIOCRUZ), Rio de Janeiro, Brazil; ^3^Instituto de Biofísica Carlos Chagas Filho, Universidade Federal do Rio de Janeiro, Rio de Janeiro, Brazil; ^4^Departamento de Análises Clínicas e Toxicológicas, Faculdade de Ciências Farmacêuticas, Universidade de São Paulo, São Paulo, Brazil; ^5^Instituto de Pesquisas Biomédicas, Hospital Naval Marcílio Dias, Marinha do Brasil, Rio de Janeiro, Brazil; ^6^Instituto do Coração Edson Saad, Hospital Universitário Clementino Fraga Filho, Universidade Federal do Rio de Janeiro, Rio de Janeiro, Brazil; ^7^National Institute of Science and Technology on Neuroimmunomodulation (INCT-NIM), Rio de Janeiro, Brazil; ^8^Faculdade de Medicina, Centro de Pesquisas em Tuberculose, Universidade Federal do Rio de Janeiro, Rio de Janeiro, Brazil; ^9^Laboratório de Imunopatologia, Instituto Oswaldo Cruz, Fundação Oswaldo Cruz (FIOCRUZ), Rio de Janeiro, Brazil

**Keywords:** Chagas disease, *Trypanosoma cruzi*, infectious heart disease, microRNA, disease biomarkers

## Abstract

Chagas cardiomyopathy is the most severe clinical manifestation of chronic Chagas disease. The disease affects most of the Latin American countries, being considered one of the leading causes of morbidity and death in the continent. The pathogenesis of Chagas cardiomyopathy is very complex, with mechanisms involving parasite-dependent cytopathy, immune-mediated myocardial damage and neurogenic disturbances. These pathological changes eventually result in cardiac myocyte hypertrophy, arrhythmias, congestive heart failure and stroke during chronic infection phase. Herein, we show that miR-208a, a microRNA that is a key factor in promoting cardiovascular dysfunction during cardiac hypertrophy processes of heart failure, has its circulating levels increased during chronic indeterminate phase when compared to cardiac (CARD) clinical forms in patients with Chagas disease. In contrast, we have not found altered serum levels of miR-34a, a microRNA known to promote pro-apoptotic role in myocardial infarction during degenerative process of cardiac injuries thus indicating intrinsic differences in the nature of the mechanisms underlying the heart failure triggered by *Trypanosoma cruzi* infection. Our findings support that the chronic indeterminate phase is a progressive phase involved in the genesis of chagasic cardiopathy and point out the use of plasma levels of miR-208a as candidate biomarker in risk-prediction score for the clinical prognosis of Chagas disease.

## Introduction

Chagas disease or American trypanosomiasis is a tropical parasitic disease caused by the protozoan *Trypanosoma cruzi* and transmitted mainly by insects of the subfamily Triatominae (Rassi et al., 2010). It is estimated that more than seven million people, especially in Mexico, Central America and South America, have Chagas disease. This has resulted in about 12,500 deaths per year since 2006. Large-scale population movements have expanded the areas where cases of Chagas disease are found, including now many countries in Europe and North America (World Health Organization, 2017). In the endemic areas, the vector consists the main mechanism of human transmission through host contact with feces and urine of infected vectors, mainly of the genera *Triatoma, Rhodnius*, and *Panstrongylus*. The insect vector acquires *Trypanosoma cruzi* parasite by feeding blood from infected animals or humans (Noireau et al., 2009).

Within the invertebrate vector, the parasite multiplies under epimastigote forms that later differentiate into trypomastigotes in the digestive tract of triatomine. The trypomastigote forms are the infecting stages of vertebrate host, being released in the feces of triatomine during bloodfeeding, and transmitted when host scratches contaminated feces into the wound (Kollien and Schaub, 2000). Once inside the host, the trypomastigote invades the cells near the inoculation, in the first line resident macrophages, where they differentiate into intracellular amastigotes (Burleigh and Andrews, 1995). The amastigotes multiply by binary division and differentiate into trypomastigotes, which are released into the bloodstream, infecting cells of various tissues, and this cycle is repeated several times, due to its ability to persist prolonged periods in the host tissues mainly as dividing amastigote forms inside tissue cells and pseudocysts by subverting humoral and cell-mediated immunity. The clinical manifestations in humans are the result of this infectious cycle and may include, at early stages of infection, symptoms such as fever, lymphadenitis, or local swelling of the biting site (Burleigh and Andrews, 1995; Kollien and Schaub, 2000; Andrade and Andrews, 2005).

After an asymptomatic acute phase in 90% of infected individuals, the infection persists and enters its chronic indeterminate phase, in which 60–80% of patients will never develop clinical symptoms, although parasitism remains in balance with host immune responses. During the intermediate phase there is a cumulative progression to diffuse myocardial damage in about 20–40% of the chronic patients developing cardiac and/or digestive problems (Teixeira et al., 2011). The pathogenesis of Chagas disease is due to an intense inflammatory lesion and fibrosis induced to the tissues and organs involved in the infectious cycle of the parasite (Machado et al., 2000; Lepletier et al., 2014). If left untreated, Chagas disease can be fatal, in most cases by damaging cardiac muscle tissue (Marin-Neto et al., 2007). The cytopathic effects induced by the parasite and the host immune responses against the intracellular amastigote harm the intramural neurons of the autonomic nervous system of the gut and heart, leading to megacolon and cardiac aneurysms, respectively (Zhang and Tarleton, 1999; Campos et al., 2016).

The nature of Chagas disease lesions induced by parasitism is intrinsically related to the perpetuation of the parasite in the affected tissues (Rassi et al., 2009). The chronicity of infection is associated with profound changes in the molecular levels of the affected organs, as it has been shown that myocardial gene expression patterns are altered in advanced forms of chronic disease (Ferreira et al., 2014). Although the molecular mechanisms underlying the myocardial gene expression are not well elucidated, studies have shown that microRNAs involved in the genetic regulation process of heart development and cardiovascular disorders have their expression disregulated in advanced forms of chagasic chronic heart disease (Eulalio et al., 2012). These studies were performed in patients with more advanced forms of chagasic heart disease and their comparisons with normal healthy individuals (Eulalio et al., 2012). However, studies in the earliest forms of chronic infection, using patients in the indeterminate phase of the disease, would allow us to explore the use of cardiac microRNAs as a potential biomarker to predict/diagnose, in the indeterminate phase of disease, the patients that will develop cardiomyopathy (20–40%).

Along these lines, we investigated herein the modulation of potential heart disease-specific miRNA biomarkers in chagasic patients at early chronic infection to be used as a risk predictor for clinical prognosis in *Trypanosoma cruzi*-induced cardiomyopathy. Our research include analysis of cross-sectional studies from chronic chagasic patients at the indeterminate (IND) as compared to cardiac (CARD) clinical forms of Chagas disease, or non-infected control individuals to profile the plasma expression of cardiac miRNA biomarkers of two extreme processes involved in the cardiovascular dysfunction. In degenerative cardiovascular diseases, microRNAs (miRNAs) are crucial regulators of cardiac function (Filipowicz et al., 2008; Boon et al., 2013). Endogenous miRNAs were shown to regulate gene expression at the post-transcriptional level either through translation repression or mRNA degradation affecting independent processes resulting in degeneration of the cardiac tissue (Callis et al., 2009).

MiR-208a is one of the most important heart-specific miRNA playing a critical role in the heart failure and has been considered as a potential biomarker of myocardial injuries (Cunha-Neto et al., 2005; Wang et al., 2013; Doka et al., 2015; Shyu et al., 2015; Zhang et al., 2017). The miR-208a is encoded by an intronic region of the alpha-cardiac muscle myosin heavy chain gene (Myh6) regulating the myosin heavy chain isoform switch (Cunha-Neto et al., 2005). Under pathophysiologic conditions, miR-208a is critical to induce arrhythmias, cardiac remodeling, expression of hypertrophy pathway components and the cardiac conduction system (Cunha-Neto et al., 2005). Studies on mice lacking miR-208a have indicated that this microRNA is required for expression of the homeodomain-only protein (HOP), which is required to modulate cardiac growth and development, in addition to GATA4 and the Gap junction alpha-5 protein (GJA5), also known as connexin 40 (Cx40). These factors are determinants in the induction of heart injuries associated with cardiac conduction (Shyu et al., 2015).

In a different aspect of the mechanisms capable of promoting heart injury, miR-34a is shown to contribute to the age-dependent deterioration of cardiac function, contributing to cardiomyocyte death following acute myocardial infarction (Filipowicz et al., 2008). Studies have identified the PPP1R10 gene (also known as PNUTS) as a direct miR-34a target, whose inhibition promotes telomere shortening and cardiomyocyte apoptosis. The anti-apoptotic role of PNUTS and its counter-regulation by age-induced expression of miR-34a has a direct effect on the processes that regulate cardiac contractile function and functional cardiac recovery after acute myocardial infarction (Filipowicz et al., 2008). In the present study we attempted to clarify whether changes in the circulating levels of miR-208a and miR-34a in the blood is associated with the development of severe cardiac clinical form of human chronic Chagas disease.

## Materials and methods

### Ethics statement

All the protocols for animal (IMPPG038-05/16) and human (CAAE No. 46502615.1.0000.5257) studies were approved by the Research Ethics Committee of Federal University of Rio de Janeiro. Experimental mice infections and assay procedures with *T. cruzi* were performed in biosafety area recommended for activities with infective stages of the parasites, in accordance with the terms of the Brazilian and international guidelines for the welfare regulations.

### Study population

Healthy volunteers and *T. cruzi* chronic chagasic patients were recruited from Chagas Cardiomyopathy clinic of Hospital Universitário Clementino Fraga Filho of Universidade Federal do Rio de Janeiro, HUCFF-UFRJ, Brazil. All individuals analyzed in this study, including chronic infected patients and non-infected individuals with ages ranging from 30 to 64 years, confirmed the diagnosis for *T. cruzi* infection in serological tests. The selected individuals included 10 seropositive cases of cardiac chronic-infected patients showed dilated cardiomyopathy diagnosed based in a detailed clinical examination, electrocardiography (ECG), and unidimensional/bidimensional echocardiography with Doppler (ECHO). Additionally, we included 10 patients in the indeterminate forms of Chagas disease, without any cardiac alterations detected. Twenty donors, age and sex matched-non-infected controls were included in the study. The research was approved by the Research Ethics Committee (CEP) and all patients signed a free and informed consent form in accordance with current legislation and the regulations of the HUCFF-UFRJ (CAAE No. 46502615.1.0000.5257).

### Quantification of human plasma levels of microRNA

Human plasma samples were collected using heparin-coated tubes and frozen at −80°C. RNA from 200 μl of human plasma were isolated with miRNeasy Serum/Plasma Kit (Qiagen) according to manufacturer instructions. Synthetic *C. elegans* miR-39 mimic (Qiagen) was spiked-in to all samples before RNA isolation to control for proper isolation. We performed miRNA expression profiling using the Taqman miRNA Assays for miR-34a (hsa-miR-34a-5p; ID number 000426), miR-208a (hsa-miR-208a-3p; ID number 000511), miR-221 (hsa-miR-221-3p; ID number 000524), miR-484 (hsa-miR-484; ID number 001821), and miR-39 (cel-miR-39; ID number 000200) in technical triplicate and the Taqman miRNA Reverse Transcription kit and Taqman Universal Master Mix II, no UNG according to the manufacturer's instructions (ThermoFisher) in a Step ONE Plus Fast Real Time PCR System (Applied Biosystems). The data were analyzed by fitting four-parameter sigmoid curves to the Rn data using the qPCR library (Ritz and Spiess, 2008) for the R statistical package version 2.922. The gene-stability measurement was determined by the optimal number of control genes required for normalization using average pairwise variation (V_n/n+1_) analysis between the normalization factors NF_n_ and NF_n+1_ by geNorm method (Vandesompele et al., 2002). An adaptation of this method was used to assay the cel-miR-39 (spike-in) for the average expression stability of reference genes, beside the non-related hsa-miR-221-3p and hsa-miR-484 endogenous genes. Based on our analysis we selected hsa-miR-221-3p and hsa-miR-484 as most stable miRNA in our samples, since they are below the recommended threshold for pairwise variation V of 0.15 (between the normalization factors NF_n_ and NF_n+1_) and used them for normalization. The comparison of the means of the normalized gene expression values between the groups was performed by a non-parametric one-way ANOVA with 1,000 unrestricted permutations, followed by *post-hoc* pair-wise comparisons with a Bonferroni adjustment using a non-parametric *t*-test, also with 1,000 permutations (Basso et al., 2009).

### Animals, infection, parasite load, detection of creatine kinase cardiac isoenzyme and intra-cardiac levels of miR-34a

Male C57BL/6 mice, aged 4–8 weeks, were housed and maintained in IMPPG-Federal University of Rio de Janeiro animal facility for experimental infection experiments. Chronic *T. cruzi* infection was performed by inoculating the 6-week-old mice intraperitoneally with 2 × 10^3^ blood-derived trypomastigote forms of Y strain of *T. cruzi*. The parasitemia was monitored by counting blood parasites using Neubauer's chambers, and animals were bled and sacrificed at 30 days post-infection (DPI). To test the creatine kinase cardiac isoenzyme (CK-MB), plasma samples were collected at 14 DPI from *T. cruzi* infected and non-infected mice, and the CK-MB activity was measured by the commercial kit CK-MB Liquiform (Labtest) as described by the manufacturer. The assays were read in a microplate spectrophotometer (SpectraMax® Plus 384 Microplate Reader, Molecular devices) allowing the analysis of small quantities of mouse plasma according to manufacturer's recommendation. The optical density at 340 nm was recorded every 1 min (A_1_) at 37°C, for 5 min (A_2_). Calibration and quality control of the equipment were performed according to the recommended protocol. To assess the intracardiac parasite load, hearts were collected at 14 DPI, minced and 100 mg of tissue were homogenized in 1 mL of TRIzol® (Invitrogen) for DNA extraction, following manufacturer's instructions. The concentrations of extracted total DNA were measured using NanoDrop-1000 (Thermo Scientific). Each PCR reaction contained 50 ng of the genomic DNA, 0.25mM *T. cruzi* 195-bp repeat DNA-specific primers: TCZ-F: 5′-GCTCTTGCCCACAAGGGTGC-3′, and TCZ-R: 5′-CCAAGCAGCGGATAGTTCAGG-3′, amplified with SYBR Green® PCR Master Mix (Qiagen) and PCR-grade H_2_O (Qiagen) on a Step One Real Time PCR System (Applied Biosystems). For loading controls, we used reactions containing 50 ng of the genomic DNA, 0.25 mM of murine-specific tumor necrosis factor-a (TNF-a) gene primers TNF-α-F: 5′-CCTGGAGGAGAAGAGGAAAGAGA-3′ and TNF-α-R: 5′-TTGAGGACCTCTGTGTATTTGTCAA-3′. To determine the intracardiac levels of mir-34a expression, high-purity miRNA was obtained from heart tissue by using standard protocol (Faragó et al., 2011). Quantitative real-time PCR analysis were performed using miScript SYBR Green PCR Kit for mouse mir-34a (Quiagen) at the same conditions as described above.

### Statistical analysis

Statistical analyses were performed with GraphPad Prism 5 software. Statistical differences between groups were compared by a non-parametrical test (Mann–Whitney Rank Sum Test). Results were expressed as mean ± standard deviation (S.D.), and differences between control and treated group were considered statistically significant when *p* ≤ 0.05.

## Results

To evaluate whether changes in the plasma-circulating levels of cardiac-specific miRNAs is correlated with Chagas heart disease severity, we first aimed at normalizing the gene-expression levels by selected stable internal control gene in order to remove any non-specific variation of the target miRNAs in our analysis. To validate the genetic expression analyzes of the RT-qPCR data we should normalize the gene expression profiles of the cardiac-specific miRNA genes that are supposed to be modulated in the process of infectious cardiopathy with respect to stably expressed endogenous reference genes not related to the disease. However, it has been demonstrated that stable microRNAs derived from plasma/serum are likely disease-specific, and miRNA control genes for Chagas disease have not yet been identified. To address this question, beside using a spike with a micro RNA control which only controls for proper isolation and processing of miRNA samples we also used irrelevant microRNA genes known to be highly regulated in our donor sample collection study, as reference genes.

**Figure 1:**
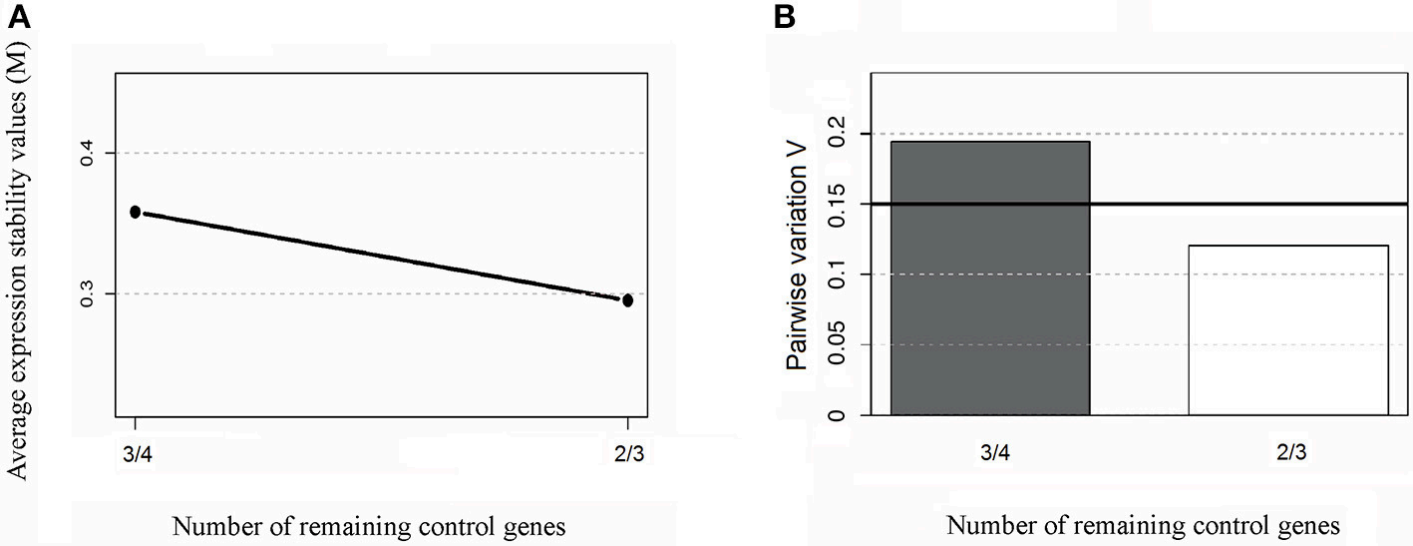
Average expression stability of endogenous miRNAs. Ranking of the gene expression stability (M) performed in all samples analyzed in the study during stepwise exclusion **(A)**. A lower *M*-value indicates more stable expression. The least stable genes are on the left using the spike-in cel-miR-39 control gene; and the most stable genes are represented on the right using the expression of endogenous hsa-miR-221-3p and hsa-miR-484 as references. The non-related microRNAs hsa-mir-221-3p and hsa-miR-484 were used for relative normalization as endogenous controls. The optimal number of control genes was determined for normalization by Pairwise variation **(B)**, as described in section Materials and Methods.

**Figure 2:**
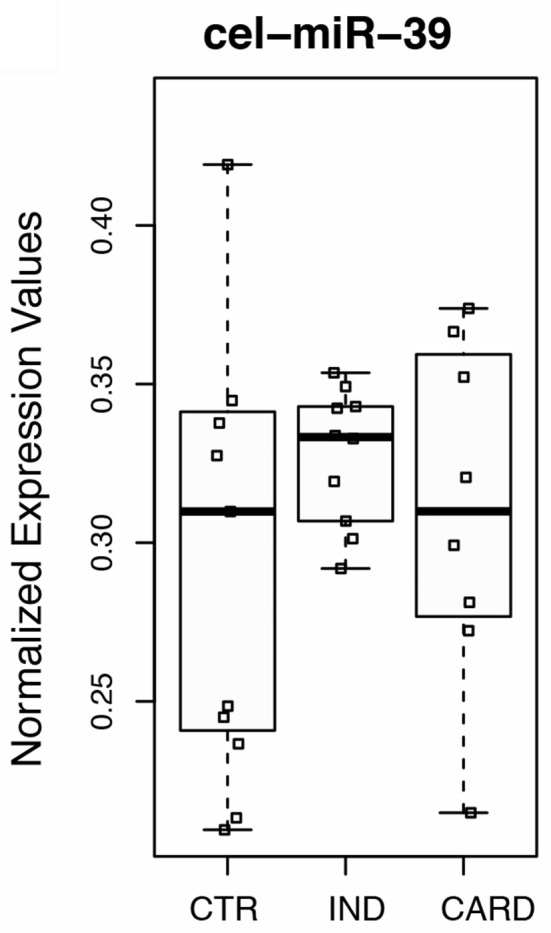
Normalyzation analysis of cardiac-specific miR-208a and miR-34a related to cel-miR-39 spike-in gene. Spike-in control using external control miRNA gene expressed in *C. elegans* (cel-miR-39) was determined to access proper sample isolation for accurate comparison between samples. Real-time quantitative PCR was performed on total miRNA purified from plasma of non-infected individuals (CTR), patients at the indeterminate (IND), and cardiac (CARD) clinical forms of Chagas disease. Twenty donors, age, and sex matched-individuals were included for each group. Groups were compared by Mann-Whitney non-parametric test.

To this end, the expression ratios of both cardiac-specific miR-208a and miR-34a were assessed as the pairwise variation with the non-related internal hsa-mir-221-3p genes and hsa-miR-484 used as endogenous controls for other infectious diseases such as Hepatitis B and breast cancer were defined from internal control gene-stability measure analysis (Hu et al., 2012; Li et al., 2015). We first performed the expression stability of two internal control non-related genes in all samples (Figure 1A) and the algorithm for the normalization of the endogenous microRNAs was more stable than using the cel-miR-39 spike-in control (Figure 1B). Based on our findings, we decided to normalize the expression profile of cardiac-miRNA genes using a geometric mean of miR-221 and miR-484 expression as described in the methods section. In fact normalyzation analysis of cardiac-specific miR-208a and miR-34a related to spike-in control did not have significant differences among the groups as determined to access proper sample isolation for comparison between samples (Figure 2).

**Figure 3:**
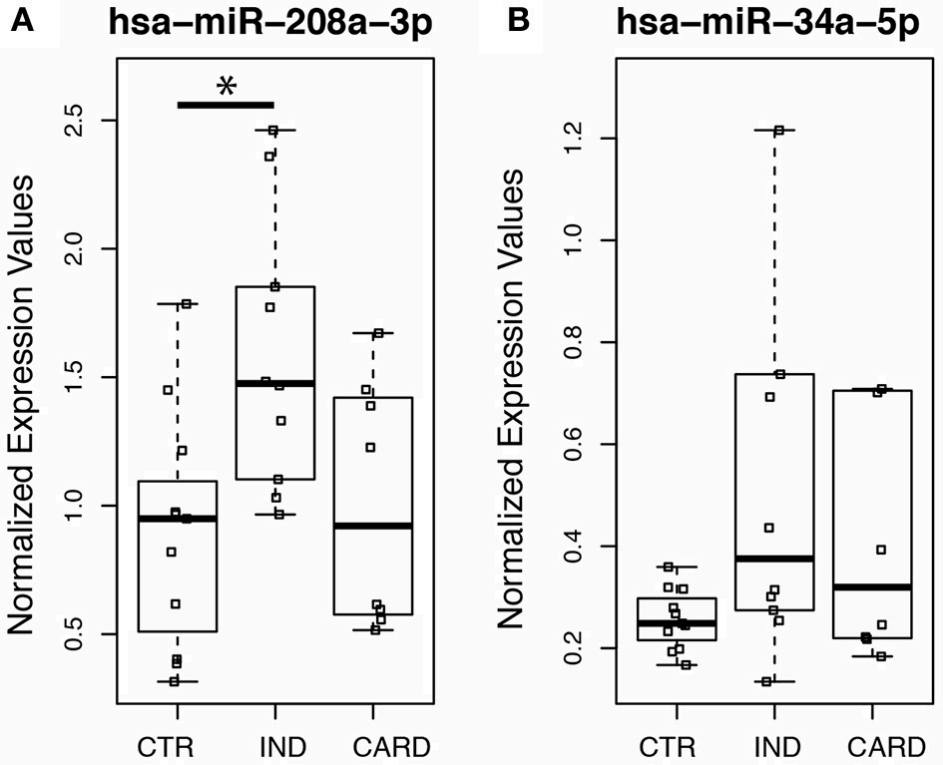
Expression profiling of the heart-specific miR-34a and mir-208a in peripheral blood from chronic chagasic patients using endogenous miRNAs. Quantitative PCR was performed on total miRNA purified from plasma of normal control individuals (CTR), patients at the indeterminate (IND), and cardiac (CARD) clinical forms of Chagas disease. Individual and median values of relative expression of **(A)** hsa-miR-208a-3p or **(B)** hsa-miR-34a-5p normalized by the geometric mean of endogenous non-related hsa-mir-221-3p and hsa-miR-484 expression. Twenty donors, age, and sex matched-individuals were included for each group. Groups were compared by Mann-Whitney non-parametric test and the differences were considered significant at ^*^p < 0.05.

Using this averaging of the control genes in order to measure expression levels accurately, we next investigate whether the myocardial damage induced by *T. cruzi* infection is associated with the expression of miR-208a, a key factor in promoting cardiac hypertrophy during cardiovascular dysfunction. Based on our results, we found significant increased levels of circulating plasma miR-208a in cross-sectional studies (Table 1) of chronic chagasic patients at the indeterminate (IND) as compared to cardiac (CARD) clinical forms of Chagas disease or non-infected control individuals (Figure 3A). However, we did not find any significant difference across the clinical groups in the expression levels of circulating miR-34a, a key regulator promoting cardiomyocyte cell death associated to heart failure (Figure 3B).

**Table 1:** Circulating plasma miRNA in cross-sectional studies of chronic chagasic patients.

	**Global *p*-value**	**log2(1ND/CTR)**	***p*-value**	**log2(CARDC/CTR)**	***p*-value**	**log2(CARDC/IND)**	***p*-value**
hsa-miR-208a-3p	0.008	0.942057053	0.005982038	0.1998333	0.923464	−0.742223753	0.18242931
hsa-miR-34a-5p	0.083	0.848200376	0.095676453	0.663184964	0.32519	−0.185015411	0.967330108

*CTR, control group; IND, indeterminate group; CARD, cardiac clinical form of Chagas disease group*.

Although our goal is to investigate the plasma levels of cardiac miRNAs as potential candidate biomarkers of risk-predicting for patients in clinical prognosis, in fact some tissue-specific miRNA do not present satisfactory circulating levels in biomarkers analyzes.

We next ruled out the possibility that the absence of this modulation is due to an intrinsic characteristic of miR-34a being differentially expressed in cardiac tissues. We then measured the levels of intracardiac expression of this miRNA using an experimental Chagas' infection model. The murine models of heart disease share at different levels some of the pathophysiological changes seen in patients with chronic heart disease, such as extra-cellular deposition of fibronectin in the heart tissue and severe alterations of the heart's electrical activity (Pereira et al., 2014). These parameters are characteristic of more advanced forms of chronic human disease, so the murine model is not useful for studying the potential pathophisiological changes observed in the human chronic indeterminate phase of Chagas disease.

Therefore, using the murine experimental model of *Trypanosoma cruzi* infection-induced cardiomyopathy, by intraperitoneally injecting C57BL/6 mice with 2 × 10^3^ bloodstream trypomastigotes of Y strain parasites, we observed a precocious blood parasitemia at day 8 post-infection (Supplementary Figure 1A), which further induced severe parasitism in the heart tissues as the infection continued (Supplementary Figure 1B). The heart parasitism intensity in this model is associated with cardiac muscle damage and can be monitored by measuring the release of CK-MB isoform of creatine kinase by heart muscle. This cardiac marker resides in the cytosol and facilitates the movement of high energy phosphates required for mitochondrial physiology.

Since CK-MB isoform has a short duration, its plasma levels can be used to access the level of heart tissue damage during the cardiopathy process (Adams et al., 1993; Pereira et al., 2014). As expected, we found a marked increase in the levels of CK-MB present in the peripheral blood circulation of *T. cruzi*-infected mice at 14 DPI (Supplementary Figure 1C). Our data indicated that this level of myocardial damage induced by *T. cruzi* infection is not associated with differences in the intracardiac expression of miR-34a as compared to non-infected controls (Supplementary Figure 1D), further indicating that the heart injury induced in the context of *T. cruzi* infection is not associated with upregulation of miR-34a pathway-dependent decline in cardiac activity. This finding corroborates the previous results showing unalterated plasma levels of miR-34a in the patients groups thus indicating that pro-apoptotic role of this cardiac miRNA is not involved in the heart injuries associated to chagas cardiomyopathy.

## Discussion

Chagas cardiomyopathy is the most severe and life-threatening manifestation of human disease caused by the protozoan parasite *Trypanosoma cruzi* (Marin-Neto et al., 2007). The knowledge of pathological changes in Chagas heart disease is largely derived from necropsy studies and endomyocardial biopsy of humans and observations in several experimental models that reasonably reproduce the various stages of the disease (Higuchi Mde et al., 1986; de Souza et al., 2001). The clinical manifestations and the tissue damage itself are closely associated with parasite multiplication and the consequent immunological reaction triggered in the parasitized myocardium (Marin-Neto et al., 2007). Thus, it is plausible to speculate that even if the parasitism is low-level, it may represent a mechanism of permanent antigenic activation and may constitute an essential pathogenic factor of immunological alteration in the chronic phase of Chagas disease (Rossi et al., 2010; Tarleton, 2015).

With the remission of parasitemia and systemic inflammatory reactions, it is believed that during the indeterminate stage the process of focal active myocarditis is more frequent, leading to the cumulative destruction of myocardial fibers, associated with microvascular disorders, intense neuronal depopulation, and the establishment of a gradual process of reparative fibrosis of cardiac tissue (Marin-Neto et al., 2007; Machado et al., 2012). Several studies using human and experimental models have shed light on the cellular and molecular mechanisms underlined in the pathogenesis of chronic manifestation of Chagas diseases. The heart infection by *T. cruzi* causes an intense inflammatory reaction resulting in leukocyte infiltration of the organ with an severely altered pattern of inflammatory-mediated factors in the infected tissues as seen by the increased expression levels of pro-inflammatory cytokines, chemokines, nitric oxide and vasoactive mediators in the cardiovascular system, with the predominance of CD8 T cells among lymphocyte populations in the chronic chagasic cardiomyopathy (Machado et al., 2012; Tarleton, 2015).

At the cellular and tissue level, the inflammatory injury of the cardiac tissue is characterized by the presence of parasitic pseudocysts associated with myonecrosis and vasculitis, with an intense accumulation of extracellular collagen affecting the function of cardiac muscle fibers (Machado et al., 2012). It has been shown that transforming growth factor-beta (TGF-β) participates in the fibrosis and heart remodeling during infection influencing the development of myocardiopathy in Chagas disease (Araújo-Jorge et al., 2002). Activation of fibrosis processes occurs through assessment of TGF-β-induced Smad2/3 and p38/ERK signaling (Zhang et al., 2006; Kolosova et al., 2011). The chagasic myocardial damage is disseminated throughout the heart, leading to electrocardiogram (ECG) abnormalities due to arrhythmias, conduction disturbances and also repolarization changes. Those alterations are representative of the widespread cardiac tissue damage (Elizari and Chiale, 1993; Machado et al., 2012). The nature of this injury process is considered an important feature of the mechanisms associated with pathogenesis of Chagasic cardiovascular disease (Machado et al., 2012).

In the present study we demonstrated that the human chronic indeterminate phase of Chagas disease results in increased levels of circulating miR-208a, an essential regulator of the genes involved in cardiac hypertrophy and fibrosis (Cunha-Neto et al., 2005; Wang et al., 2013; Doka et al., 2015; Shyu et al., 2015; Zhang et al., 2017). MiR-208a is encoded by an intronic region of the Myh6 gene that encodes α-myosin heavy chain, the prevalent heavy-chain contractile protein in the developed adult heart (Cunha-Neto et al., 2005). Alterations in the miR-208a expression levels are frequently associated with pathological heart dysfunctions, such as hypertrophy, fibrosis, arrhythmias, contractile dysfunction and conduction abnormalities (Cunha-Neto et al., 2005; Wang et al., 2013; Doka et al., 2015; Shyu et al., 2015; Zhang et al., 2017). Indeed, overexpression of miR-208a is associated with coronary heart disease (Zhang et al., 2017), and its therapeutic inhibition leads to recovery of cardiac function during heart disease (Doka et al., 2015). It has been shown that transforming growth factor-β (TGF-β) activates mirR-208a to regulate hypertrophic-related genes and the effect of neutralizating TGF-β antibody attenuates miR-208a induced-expression in cardiomyocyte hypertrophy (Wang et al., 2013).

In fact the TGF-β signaling pathway has been shown to potentiate *T. cruzi* infection and heart damage in both human and experimental murine models (Araújo-Jorge et al., 2002, 2008, 2012; Waghabi et al., 2009). This suppressor cytokine is implicated in several aspects of host-parasite interplay. TGF-β is responsible for disarming the intracellular defense responses of *T. cruzi*-infected cells, thus potentiating the parasitic burden not only on macrophages but also on cardiac fibroblasts and cardiomyocytes (Araújo-Jorge et al., 2012); regulate the pro-inflammatory immune responses against *T. cruzi* (Gutierrez et al., 2009); and induction of fibrosis and heart injury during the chronicity of the disease (Araújo-Jorge et al., 2002, 2008; Waghabi et al., 2009). Indeed, circulating levels of TGF-β progressively increase during the chronicity of indeterminate stages for the cardiac, presenting a correlation with cardiac dysfunction and progressive fibrosis in Chagas disease (Araújo-Jorge et al., 2002). The regulation of targets by miRNAs is subject to various levels of control, and recent developments have shown that their targets can reciprocally control the level and function of miRNAs (Pasquinelli, 2012). Our data show that during the indeterminate phase of chronic infection, circulating levels of miR-208a are significantly increased. It is possible that there is a mutual regulation of miR-208a and its target genes, preceding the process of progressive fibrosis in Chagas disease during chronic infection.

Our findings therefore suggest a participation of the miR-208a in the early-onset events responsible for activation of the fibrosis and cardiac dysfunction processes in Chagas disease. Furthermore, our findings demonstrate that active myocardial inflammation promoted by *T. cruzi* infection does not yield altered levels of circulating miR-34a. MiR-34a negatively regulates the transcription of the anti-apoptotic factor PNUTS, and its response results in the induction processes of cardiac tissue degeneration (Filipowicz et al., 2008). Recent works have shown that miR-34a is an inducer of cardiomyocyte apoptosis, and its activation is determinant in cardiac senescence processes resulting in the induction of myocardial infarction processes (Filipowicz et al., 2008; Matsumoto et al., 2013). These findings suggest the participation of different mechanisms and signaling pathways of cardiac injury processes in *T. cruzi*-induced cardiomyopathy.

At present, the techniques of risk prediction in patients in the indeterminate phase of Chagas disease are based on the detection of cardiac wall motion abnormalities associated with functional and electrical defects. The presence of echocardiographic abnormalities in association with high interleukin-6 concentrations as a marker of myocardial injury has been highly predictive to screen patients for risk stratification (López et al., 2006). In this line, our findings demonstrating increased expression levels of circulating miR-208a during chronic indeterminate phase of Chagas disease should call attention to its predictive marker to identify patients at risk of developing chagasic heart disease. Subsequent translational studies should determine the levels of miR-208a in the course of chronic infection in order to determine its use as candidate biomarkers to be used in risk-prediction score for patients with Chagas cardiomyopathy.

## Author contributions

AM: Conceived and designed the experiments; LL-L, AG, JGN, and LC: Performed the experiments; LL-L, AG, JG-N, and LC: Analyzed the data; LF, CF, EF, SC, RA, RP, WS, and AM: Contributed reagents, materials, analysis tools; AG, RP, and AM: Wrote the paper.

### Conflict of interest statement

The authors declare that the research was conducted in the absence of any commercial or financial relationships that could be construed as a potential conflict of interest.

## Supplementary material

Supplementary Figure 1 Intra-cardiac levels of miR-34a expression in *T. cruzi*-induced myocarditis. **(A)** C57BL/6 mice were infected via *i.p*. with 2 × 10^3^ blood-derived trypomastigote forms of Y strain of *T. cruzi* and the parasitemia for each mouse was determined during infection. The values are represented as the mean ± the standard deviation (*SD*) (*n* = 5). **(B)** DNA was extracted from heart tissue at 14 days post-infection and the parasite load was evaluated by quantitative PCR. **(C)** CK-MB levels were analyzed from serum at 14 DPI by enzymatic activity at 37°C. **(D)** MicroRNA-34a levels were measured from serum from non-infected control and *T. cruzi*-infected animals at 14 days post-infection by quantitative RT-PCR. Data are means ± *SD*. and represent the results of three independent experiments performed with 5 mice per treatment. Differences between between the groups were considered significant ^***^*p* < 0.001.
